# Prevalence estimation of ATTRv in China based on genetic databases

**DOI:** 10.3389/fgene.2023.1126836

**Published:** 2023-04-13

**Authors:** Zheng Yongsheng, Sun Chong, Liu Bingyou, Hu Jianian, Chen Haofeng, Zhao Chongbo, Victor Wei Zhang, Lin Jie

**Affiliations:** ^1^ Department of Neurology, Huashan Hospital Fudan University, Shanghai, China; ^2^ Amcarelab Genetic Commercial Company, Guangzhou, China

**Keywords:** amyloid transthyretin (ATTR), ChinaMap, GnomAD, prevalence, genetic database

## Abstract

**Introduction:** Amyloid transthyretin (ATTR) is divided into either hereditary (ATTRv) or sporadic (ATTRwt) and ATTRv is a rare hereditary disease transmitted as an autosomal dominant manner. Its global prevalence is traditionally estimated as 5,000 to 10,000 persons. However, it may be underestimated and the exact prevalence of ATTRv in China mainland remains unknown.

**Methods:** The Genome Aggregation database (gnomAD) database (containing 125,748 exomes) and two genomic sequencing databases——China Metabolic Analytics Project (ChinaMAP) (containing 10588 individuals) and Amcarelab gene database (containing 45392 exomes), were integrated to estimate the prevalence of ATTRv in the world and mainland Chinese populations. Pathogenic variants allele frequency and the prevalence of ATTRv was calculated.

**Results:** Six variants, counting 470 alleles, were defined as pathogenic variants in gnomAD. The prevalence of ATTRv in the world population was 57.4/100,000. Two variants (2 allele counts) and 15 variants (34 individuals) were defined as pathogenic variants in the ChinaMAP database and the Amcarelab exome database, respectively. Thus, the estimated prevalence interval of ATTRv in mainland China was 18.9/100,000-74,9/100,000.

**Conclusion:** The present study demonstrated that the previous prevalence was greatly underestimated using traditional methods. Therefore, raising awareness of the disease is essential for recognizing ATTRv in its early stage.

## Introduction

Amyloid transthyretin (ATTR) amyloidosis is characterized by pathologic accumulation of extracellular protein arising from unstable transthyretin (TTR) tetramers, which dissociate into monomers that misfold, aggregate, and form insoluble fibrils that are resistant to proteolysis (Finsterer et al., 2019). TTR misfolding can lead to two distinct forms of amyloidosis: hereditary (ATTRv) and wild-type (ATTRwt) (Yamamoto and Yokochi, 2019). ATTRv is a rare hereditary disease transmitted as an autosomal dominant manner (Adams et al., 2016; Adams et al., 2021a). It is caused by mutations in the transthyretin gene located on chromosome 18 (18q12.1). There is considerable heterogeneity in the clinical presentation of ATTRv, ranging from primarily cardiac, primarily neuropathic, or other progressive multisystem disorders (Rapezzi et al., 2010). ATTRv usually proves fatal within 7–12 years from the onset of symptoms, causing a heavy economic burden for families and society (Takeshima et al., 2019; Galan et al., 2021).

ATTRv is an underdiagnosed disease and the true incidence and prevalence are currently unknown (Obi et al., 2022). Its global prevalence is traditionally and somewhat anecdotally estimated as 5,000 to 10,000 persons but only several countries in Europe and Japan have relatively exact prevalence (Sousa et al., 1995; Kato-Motozaki et al., 2008; Dardiotis et al., 2009; Ines et al., 2018; Lauppe et al., 2021). In epidemic regions such as Portugal, Sweden, and Japan, the prevalence varies from 10/100,000 to 100/100,000 people. Recently, the prevalence of transthyretin familial amyloid polyneuropathy (ATTR-FAP) in China was estimated to be approximately 2000 (range 435–10,134) [1,347.7 million persons in total] (Schmidt et al., 2018). However, the prevalence estimation and information supporting prevalence calculations were extracted from records yielded by reference-databases searches, conference proceedings and published cases or case series, which certainly had it underestimated.

Previous prevalence estimation was given using traditional methods, namely, case reports, epidemiological surveys and patient registries (Ikeda et al., 2002; Ines et al., 2018). These traditional methods are prone to underestimations due to diagnostic bias and small sample size. In recent years, population-based genomic sequencing projects provided a new genetic perspective on the estimation of disease-related prevalences (Lahuerta Pueyo et al., 2019). To date, several studies have employed publicly available large-population databases to infer pathogenicity, penetrance or prevalence of rare diseases (Rutten et al., 2016; Lahuerta Pueyo et al., 2019; Liu et al., 2019). Herein, we utilized an integrated world population and Chinese population genomic sequencing databases to reevaluate the ATTRv prevalence in the mainland Chinese population.

## Materials and methods

### The Genome Aggregation database

GnomAD contained 125,748 exomes from unrelated individuals and from different populations, sequenced as part of various disease-specific and population genetic studies. All *TTR* (canonical transcript ENST00000237014) variants were selected from the gnomAD version 2.1.1 (http://gnomad.broadinstitute.org/). The analysis of the gnomAD data was carried out from June 2022 to August 2022. In terms of populations, all variants analyzed were classified according to the diverse ethnicities defined in the gnomAD database. The population classifications were as follows: non-Finnish European, African/African American, Latino, Finnish, Ashkenazi Jewish, East Asian, South Asian and other. Population in mainland China was not included in the gnomAD.

### The China metabolic analytics project database

The ChinaMAP was based on cohort studies across diverse regions and ethnic groups in China. ChinaMAP (beta version, 2020-03) provided allele frequencies data of variants identified in the Chinese population (*n* = 10588). It randomly selected participants from 8 ethnic groups (Han, Hui, Manchu, Miao, Mongolian, Yi, Tibetan and Zhuang) across 27 provinces of China without biased selection or filtration (Cao et al., 2020). Therefore, the ChinaMAP database may be considered as a genetic database for the general Chinese population. All *TTR* (transcript NM_000371.3) variants were extracted from www.mBiobank.com on August 2022.

### The Amcarelab gene database

The Amcarelab gene database was provided by the Amcarelab genetic commercial company. It is composed of 45392 individuals who were suspected to have gene related disorders by medical practitioners from different clinical specialties. Therefore, this database included a subpopulation with negative results from genetic testing as well as patients with definite genetic disorders. All *TTR* (transcript NM_000371.4) variants were extracted on June 2022.

### Data analysis and variant annotation

The present study is focused on missense mutations, genetic variations that result in the substitution of one amino acid with another. The disease-associated variants analyzed in this study specifically affect protein folding, which leads to the accumulation of misfolded proteins, rather than the truncation of protein function. For this reason, the study excluded splicing variants, stop codons, and frameshift changes, as these variants may lead to the production of truncated or non-functional proteins. Additionally, intronic variants, variants in non-coding regions, and synonymous variants were not considered in the study. Following the recommendations of American College of Medical Genetics and Genomics (ACMG), the variants were divided into the following categories: pathogenic, likely pathogenic, uncertain significance, likely benign, benign. In present study, pathogenic and likely pathogenic variants were further defined as disease-causing variant. The calculation of the prevalence of disease-causing variants is our main goal.

## Results

### Allele frequency of pathogenic variants in gnomAD

**FIGURE 1:**
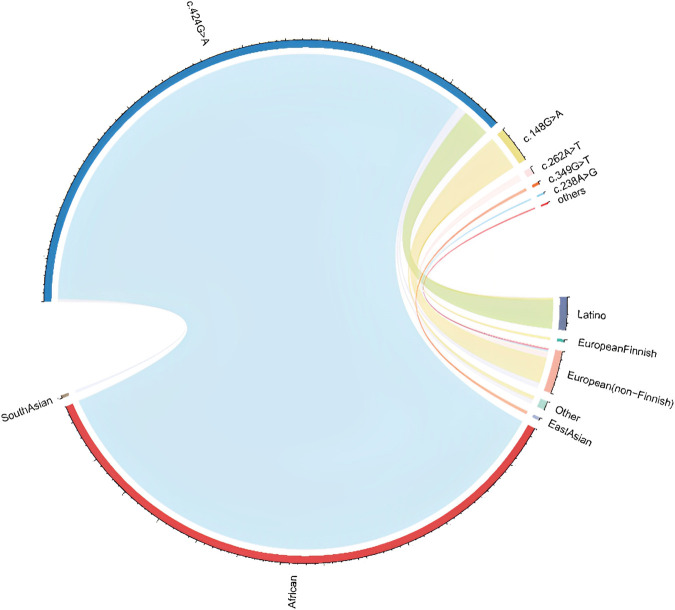
Population and pathogenic variant correlation in gnomAD Abbreviations: The ethical information was achieved from gnomAD database. The line connecting represent the pathogenic variant present in the ethnic population. The thicker the line, the higher the proportion of the corresponding variant in that ethnic population.

About 66 missense variants were found in the *TTR* gene, with an average of 125,780 exomes studied. According to our definition, six disease-causing variants (including 470 alleles count) were identified and their minor allelic frequency (MAF) was described in Table 1. There were 3 homozygotes identified. The disease-causing amyloidogenic variants MAF was 0.00186 and so the overall prevalence was 371.3/100,000. This is about 4-fold–40-fold higher than what would be expected based on the current epidemic regions prevalence estimation of 10/100,000 (actually from 10/100,000 to 100/100,000) (Ikeda et al., 2002; Kato-Motozaki et al., 2008). This huge prevalence gap between the estimation based on gene databases and traditional methods led us to conduct a further investigation. At first, we analyzed the relationship between population ethnicities and variants. Interestingly, we found a strong relation between the African population and the variant c.424G>A (p. (Val142Ile)) (Figure 1). Among the amyloidogenic *TTR* pathogenic variants, the point change c.424G>A is unusual for its overwhelmingly predominant occurrence in individuals of documented African descent. Previous studies have shown that the c.424G>A variant was reported in the European population with an allele frequency of 0.00004, while in the African population it was reported with an allele frequency of 0.016 (Lahuerta Pueyo et al., 2019). According to more recent data, the worldwide prevalence of this variant ranges from 0.3% to 1.6% in the general population. However, studies have reported higher prevalence rates of the c.424G>A variant among people of African descent, ranging from 1.1% to 9.8%. It is important to note that the prevalence of the c.424G>A variant in a specific region is highly dependent on the percentage of people with African ancestry residing in that region (Chandrashekar et al., 2021). For its predominance in African/African-American, we further classified pathogenic missense variants found in gnomAD into two groups: African/African-American population and non-African. Then, the allele counts of the African/African-American population group based on gnomAD was 402 in 12487 exomes, and the allele frequency was 0.016, which was consistent with the prevalence previously reported. At last, the allele counts of the non-African group was 65 in 113261 exomes and the allele frequency was 0.0000287 (prevalence: 57.4/100,000).

**TABLE 1:** Variants in the *TTR* gene classified as disease-causing variants from the gnomAD database global population.

Genomic location (GRCh37)	Transcript	Protein ID	Variant	Protein change	Allele count	Homozygote count	Allele frequency	Variants categories
chr18: 29172937	NM_000371.4	NP_000362.1	c.148G>A	p.V50M	26	0	0.000103	Pathogenic
chr18: 29175120	NM_000371.4	NP_000362.1	c.238A>G	p.T80A	1	0	0.00000398	Pathogenic
chr18: 29175132	NM_000371.4	NP_000362.1	c.250T>C	p.F84L	1	0	0.00000398	Pathogenic
chr18: 29175144	NM_000371.4	NP_000362.1	c.262A>T	p.I88L	5	0	0.0000199	Likely pathogenic
chr18: 29178543	NM_000371.4	NP_000362.1	c.349G>T	p.A117S	2	0	0.00000795	Likely pathogenic
chr18: 29178618	NM_000371.4	NP_000362.1	c.424G>A	p.V142I	435	3	0.00174	Likely pathogenic

### Prevalence estimation based on the ChinaMAP database

Analysis of the 10588 Chinese individuals identified 32 *TTR* missense mutation variants (Supplementary Material S1). Importantly, 2 variants (allele counts: 2), c.148 > A (p.Val50Met) and c.424G>A (p.Val142Ile) were classified as disease-causing variants according to this study. The allele frequency of disease-causing variants in the *TTR* within the Chinese population was estimated to be 0.0000944 (prevalence: 18.9/100,000).

### Prevalence estimation based on the Amcarelab exome database

Excluding synonymous, non-sense, splice variants, intron variants and variants in non-coding regions, analysis of the 45392 exomes identified 107 missense *TTR* variants **(** Supplementary Material S2 **)** and 15 variants were pathogenic and likely pathogenic (34 individuals) (Table 2). Thus, the prevalence of disease-causing variants in Chinese population was estimated to be 34 in 45392, namely 74.9/100,000. Herein, the estimated prevalence interval of ATTRv in mainland China was 18.9∼74.9/100,000, based on the public exome, ChinaMAP, and the commercial exome databases.

**TABLE 2:** Variants in the *TTR* gene classified as pathogenic variants from the Amcarelab database.

Genomic location (GRCh37)	Transcript	Protein ID	Variant	Protein change	Individual count	Number of homozygotes	Variants categories	Previously reported
chr18: 29172902	NM_000371.4	NP_000362.1	c.113A>G	p.D38G	4	0	Likely pathogenic	Yes
chr18: 29172937	NM_000371.4	NP_000362.1	c.148G>A	p.V50M	8	0	Pathogenic	Yes
chr18: 29172954	NM_000371.4	NP_000362.1	c.165G>T	p.K55N	1	0	Likely pathogenic	Yes
chr18: 29172988	NM_000371.4	NP_000362.1	c.199G>A	p.G67R	1	0	Likely pathogenic	Yes
chr18: 29175092	NM_000371.4	NP_000362.1	c.210T>A	p.S70R	1	0	Likely pathogenic	Yes
chr18: 29175096	NM_000371.4	NP_000362.1	c.214T>C	p.S72P	1	0	Likely pathogenic	Yes
chr18: 29175103	NM_000371.4	NP_000362.1	c.221A>C	p.E74A	1	0	Likely pathogenic	Yes
chr18: 29175106	NM_000371.4	NP_000362.1	c.224T>G	p.L75R	1	0	Likely pathogenic	Yes
chr18: 29175123	NM_000371.4	NP_000362.1	c.241G>A	p.E81K	3	0	Likely pathogenic	Yes
chr18: 29175172	NM_000371.4	NP_000362.1	c.290C>T	p.S97F	1	0	Likely pathogenic	Yes
chr18: 29175193	NM_000371.4	NP_000362.1	c.311T>A	p.I104N	1	0	Likely pathogenic	Yes
chr18: 29175207	NM_000371.4	NP_000362.1	c.325G>A	p.E109K	3	0	Likely pathogenic	Yes
chr18: 29178543	NM_000371.4	NP_000362.1	c.349G>T	p.A117S	5	0	Likely pathogenic	Yes
chr18: 29178595	NM_000371.4	NP_000362.1	c.401A>G	p.Y134C	1	0	Likely pathogenic	Yes
chr18: 29178618	NM_000371.4	NP_000362.1	c.424G>A	p.V142I	2	0	Likely pathogenic	Yes

### Prevalence estimation in the previous reported studies and in present study

Table 3 showed the prevalence of ATTRv in previous reports. In epidemic regions such as Portugal, Japan, and Sweden, the prevalence ranges from 1.1/100,000 to 151/100,000 according to different estimation methods. In non-epidemic regions, the prevalence various from 0.433/100,000 to 3.72/100,000. Specially, ATTRv prevalence in Saudi was estimated to 21.5/100,000 using genome sequencing database.

**TABLE 3:** Previous ATTRv prevalence estimations.

Country	Disease classification	Prevalence	Method	Published year	References
Northern Sweden	ATTR-FAP	104/100,000	Traditional methods	1976	Andersson (1976)
Japan	ATTRv	15/100,000	Traditional methods	2002	Ikeda et al. (2002)
Japan	ATTRv	1.1/100,000	Traditional methods	2008	Kato-Motozaki et al. (2008)
Cyprus	ATTR-FAP	3.72/100,000	Traditional methods	2009	Dardiotis et al. (2009)
Northern Portugal	ATTR-FAP	151/100,000	Traditional methods	1995	Sousa et al. (1995)
Austria	ATTRv	0.5/100,000	Traditional methods	2019	Auer-Grumbach et al. (2020)
Italy	ATTRv	0.433/100,000	Traditional methods	2020	Russo et al. (2020)
Saudi	ATTRv	21.5/100,000	Genome database	2021	Abouelhoda et al. (2021)
Worldwide	ATTR-FAP	10186-38,468 individuals	Traditional methods	2017	Schmidt et al. (2018)
Worldwide	ATTRv	57.4/100,000	Genome database	—	Present study
China	ATTRv	18.9–74.9/100,000	Genome database	—	Present study

ATTRv, transthyretin amyloidosis; ATTR-FAP, transthyretin familial amyloid polyneuropathy; Traditional methods refers to case reports, epidemiological surveys, and patient registries.

## Discussion

This is the first study to identify and investigate the prevalence of disease-causing *TTR* variants in the Chinese mainland population. The estimated prevalence interval of ATTRv was 18.9–74.9/100,000 based on the public exome ChinaMAP and commercial exome databases. The lower limit of the prevalence interval was as high as or near 20-fold higher than that in Japan (prevalence: 1/100,000) concluded by traditional epidemiological methods (Kato-Motozaki et al., 2008). Our results suggested that ATTRv in the Chinese was observed much more prevalent than previously recognized.

To determine whether the prevalence in China is high, we estimated the prevalence of different populations and various cohorts using gnomAD data. The gnomAD database consisted of eight ethnic groups but not all populations were equally represented, with the European population being the most represented and the Asian and Latino the least represented (Collins et al., 2020). Noteworthy, these databases did not contain the Chinese population. Comparisons to other population may aid in determine the prevalence gap of a number of disorders that exist between the Chinese and world populations. Not surprisingly, the ATTRv prevalence was 57.4 per 100,000 persons estimated by the genomic sequencing projects approach. It was equal to or even higher than that in epidemic regions such as Portugal, Sweden, and Japan, whose prevalence were calculated by case reports, epidemiological surveys and patient registries. A recent study using a national population-based exome sequence database reported the prevalence of 21.5 per 100,000 persons in Saudi (Abouelhoda et al., 2021), an Asian country that was not reported as an ATTRv epidemic region. This further supported that the true prevalence of ATTRv was much higher than that reported in previous studies.

There are four plausible explanations for the unexpected high prevalence: 1) the pathogenic variants in the ChinaMAP and Amcarelab databases differ from those found in ATTRv in reality and variants were not pathogenic but were polymorphisms, 2) all of the individuals with a *TTR* pathogenic variant in the ChinaMAP and Amcarelab databases have an undiagnosed or unreported classical *TTR* variant; 3) a late onset, mild *TTR* phenotype which was less readily diagnosed, may be much more prevalent than recognized to date. However, it is highly unlikely that any of the 15 disease-causing amyloidogenic *TTR* variants found in ChinaMAP and Amcarelab databases were polymorphisms as most the 15 pathogenic variants have been previously reported in *TTR* pedigrees. Taking the results from the gnomAD and Saudi databases into consideration, low prevalence estimation from previous traditional methods may be from penetrance *TTR* variability, misdiagnosis or missed diagnosis, delayed diagnosis and/or for other reasons (Rapezzi et al., 2010).

A high prevalence highlights the importance of diagnosing ATTRv clinically at an early stage. Recent years have seen the description of a series of novel and effective therapies for ATTRv. Tafamidis, patisiran, inotersen as well as the most promising NTLA-2001 have shown great benefits for patients with ATTRv. Specifically, these benefits are better received when ATTRv is diagnosed at an early stage of disease (Benson et al., 2018; Lamb and Deeks, 2019; Adams et al., 2021b; Gillmore et al., 2021). It challenges and encourage clinical practitioners to raise awareness and have a deeper and broader understanding of ATTRv because early diagnosis of amyloidosis remains an elusive goal that requires education of both physicians and patients (Wechalekar et al., 2016). Further examinations or even gene sequencing must be performed when there is any suspicion of hereditary amyloidosis with initial signs and symptoms, to enter the screening process for amyloidosis and improve early diagnosis (Conceicao et al., 2019).

Our study has several limitations. First, in the present study, only “pathogenic” and “likely pathogenic” variants were defined as disease-causing variants. In fact, some variants in the “uncertain significance” group may also be disease-causing. Thus, the prevalence of ATTRv may be even higher. Secondly, penetrance analysis of the *TTR* gene could not be determined. Lastly, the characteristics of the Chinese variants and the relationship between variants and clinical characteristics were not analyzed.

In conclusion, we confirmed the high prevalence of ATTRv in the Chinese mainland population, demonstrating that the previous prevalence was greatly underestimated obtained using traditional methods. Therefore, raising awareness of the disease is essential for recognizing ATTRv in its early stage.

## Data Availability

The raw data supporting the conclusions of this article will be made available by the authors, without undue reservation.

## Abbreviations

ACMG, American College of Medical Genetics and Genomics; ATTR, amyloid transthyretin; ATTR-FAP, transthyretin familial amyloid polyneuropathy; ATTRv, hereditary amyloid transthyretin; ATTRwt, wild-type amyloid transthyretin; ChinaMAP, China Metabolic Analytics Project; GnomAD, Genome Aggregation database; HGVS, Human Genome Variation Society; TTR, transthyretin.
