# Supplementary material for: Prevalence estimation of ATTRv in China based on genetic databases
Source: Front Genet. 2023 Apr 13;14:1126836. doi: 10.3389/fgene.2023.1126836 (PMC10133693; doi:10.3389/fgene.2023.1126836)
Supplement: Supplementary file 1 [file DataSheet2.PDF]

Chromosome	Gene	Transcript	Protein ID	Transcript Consequence	Ptein Consequence	Variant classification
chr18	TTR	NM_000371.4	NP_000362.1	c.114T>C	p.D38D	3
chr18	TTR	NM_000371.4	NP_000362.1	c.119T>C	p.V40A	3
chr18	TTR	NM_000371.4	NP_000362.1	c.120C>T	p.V40V	3
chr18	TTR	NM_000371.4	NP_000362.1	c.13C>T	p.R5C	3
chr18	TTR	NM_000371.4	NP_000362.1	c.148G>A	p.V50M	1
chr18	TTR	NM_000371.4	NP_000362.1	c.14G>A	p.R5H	3
chr18	TTR	NM_000371.4	NP_000362.1	c.160A>C	p.R54R	3
chr18	TTR	NM_000371.4	NP_000362.1	c.162A>G	p.R54R	3
chr18	TTR	NM_000371.4	NP_000362.1	c.170C>A	p.A57D	3
chr18	TTR	NM_000371.4	NP_000362.1	c.207C>G	p.T69T	3
chr18	TTR	NM_000371.4	NP_000362.1	c.225G>C	p.L75L	3
chr18	TTR	NM_000371.4	NP_000362.1	c.302C>T	p.A101V	3
chr18	TTR	NM_000371.4	NP_000362.1	c.315C>A	p.S105S	3
chr18	TTR	NM_000371.4	NP_000362.1	c.330T>G	p.H110Q	3
chr18	TTR	NM_000371.4	NP_000362.1	c.347C>G	p.T116R	3
chr18	TTR	NM_000371.4	NP_000362.1	c.354C>T	p.N118N	3
chr18	TTR	NM_000371.4	NP_000362.1	c.357C>G	p.D119E	3
chr18	TTR	NM_000371.4	NP_000362.1	c.360C>T	p.S120S	5
chr18	TTR	NM_000371.4	NP_000362.1	c.361G>A	p.G121S	3
chr18	TTR	NM_000371.4	NP_000362.1	c.370C>T	p.R124C	3
chr18	TTR	NM_000371.4	NP_000362.1	c.371G>A	p.R124H	3
chr18	TTR	NM_000371.4	NP_000362.1	c.385G>A	p.A129T	3
chr18	TTR	NM_000371.4	NP_000362.1	c.390G>A	p.L130L	3
chr18	TTR	NM_000371.4	NP_000362.1	c.417G>A	p.T139T	5
chr18	TTR	NM_000371.4	NP_000362.1	c.424G>A	p.V142I	2
chr18	TTR	NM_000371.4	NP_000362.1	c.429C>T	p.T143T	3
chr18	TTR	NM_000371.4	NP_000362.1	c.431A>G	p.N144S	3
chr18	TTR	NM_000371.4	NP_000362.1	c.62G>A	p.G21A	3
chr18	TTR	NM_000371.4	NP_000362.1	c.62G>C	p.G21D	3
chr18	TTR	NM_000371.4	NP_000362.1	c.69G>A	p.T23T	3

chr18	TTR	NM_000371.4	NP_000362.1	c.76G>A	p.G26S	3
chr18	TTR	NM_000371.4	NP_000362.1	c.7T>C	p.S3P	3
