# Supplementary material for: Prevalence estimation of ATTRv in China based on genetic databases
Source: Front Genet. 2023 Apr 13;14:1126836. doi: 10.3389/fgene.2023.1126836 (PMC10133693; doi:10.3389/fgene.2023.1126836)
Supplement: Supplementary file 2 [file DataSheet1.PDF]

Chromosome	Gene	Transcript	Protein ID	Transcript Consequence	Protein Consequence	Variant classification
chr18	TTR	NM_000371.4	NP_000362.1	c.100G>A	p.V34I	3
chr18	TTR	NM_000371.4	NP_000362.1	c.105A>T	p.K35N	3
chr18	TTR	NM_000371.4	NP_000362.1	c.10C>G	p.H4D	3
chr18	TTR	NM_000371.4	NP_000362.1	c.113A>G	p.D38G	2
chr18	TTR	NM_000371.4	NP_000362.1	c.115G>C	p.A39P	3
chr18	TTR	NM_000371.4	NP_000362.1	c.11A>C	p.H4P	3
chr18	TTR	NM_000371.4	NP_000362.1	c.13C>T	p.R5C	3
chr18	TTR	NM_000371.4	NP_000362.1	c.142G>C	p.V48L	3
chr18	TTR	NM_000371.4	NP_000362.1	c.148G>A	p.V50M	1
chr18	TTR	NM_000371.4	NP_000362.1	c.14G>A	p.R5H	3
chr18	TTR	NM_000371.4	NP_000362.1	c.14G>T	p.R5L	3
chr18	TTR	NM_000371.4	NP_000362.1	c.165G>T	p.K55N	2
chr18	TTR	NM_000371.4	NP_000362.1	c.169G>A	p.A57T	3
chr18	TTR	NM_000371.4	NP_000362.1	c.170C>A	p.A57D	3
chr18	TTR	NM_000371.4	NP_000362.1	c.17T>C	p.L6P	3
chr18	TTR	NM_000371.4	NP_000362.1	c.199G>A	P.G67R	2
chr18	TTR	NM_000371.4	NP_000362.1	c.19C>A	p.L7I	3
chr18	TTR	NM_000371.4	NP_000362.1	c.203A>C	p.K68T	3
chr18	TTR	NM_000371.4	NP_000362.1	c.203A>G	p.K68R	3
chr18	TTR	NM_000371.4	NP_000362.1	c.204A>C	p.K68N	3

chr18	TTR	NM_000371.4	NP_000362.1	c.204A>T	p.K68N	3
chr18	TTR	NM_000371.4	NP_000362.1	c.205A>G	p.T69A	3
chr18	TTR	NM_000371.4	NP_000362.1	c.205A>T	p.T69S	3
chr18	TTR	NM_000371.4	NP_000362.1	c.208A>C	p.S70R	3
chr18	TTR	NM_000371.4	NP_000362.1	c.208A>T	p.S70C	3
chr18	TTR	NM_000371.4	NP_000362.1	c.209G>T	p.S70I	3
chr18	TTR	NM_000371.4	NP_000362.1	c.210T>A	p.S70R	2
chr18	TTR	NM_000371.4	NP_000362.1	c.211G>C	p.E71Q	3
chr18	TTR	NM_000371.4	NP_000362.1	c.212A>C	p.E71A	3
chr18	TTR	NM_000371.4	NP_000362.1	c.212A>T	p.E71V	3
chr18	TTR	NM_000371.4	NP_000362.1	c.213G>C	p.E71D	3
chr18	TTR	NM_000371.4	NP_000362.1	c.214T>C	p.S72P	2
chr18	TTR	NM_000371.4	NP_000362.1	c.215C>A	p.S72Y	3
chr18	TTR	NM_000371.4	NP_000362.1	c.218G>C	p.G73A	3
chr18	TTR	NM_000371.4	NP_000362.1	c.221A>C	P.E74A	2
chr18	TTR	NM_000371.4	NP_000362.1	c.222G>T	p.E74D	3
chr18	TTR	NM_000371.4	NP_000362.1	c.224T>G	p.L75R	2
chr18	TTR	NM_000371.4	NP_000362.1	c.226C>T	p.H76Y	3
chr18	TTR	NM_000371.4	NP_000362.1	c.22C>T	p.L8F	3
chr18	TTR	NM_000371.4	NP_000362.1	c.230G>A	p.G77E	3
chr18	TTR	NM_000371.4	NP_000362.1	c.235A>T	p.T79S	3
chr18	TTR	NM_000371.4	NP_000362.1	c.238A>T	p.T80S	3
chr18	TTR	NM_000371.4	NP_000362.1	c.239C>T	p.T80TI	3

chr18	TTR	NM_000371.4	NP_000362.1	c.241G>A	P.E81K	2
chr18	TTR	NM_000371.4	NP_000362.1	c.241G>C	p.E81Q	3
chr18	TTR	NM_000371.4	NP_000362.1	c.243G>T	p.E81D	3
chr18	TTR	NM_000371.4	NP_000362.1	c.244G>C	p.E82Q	3
chr18	TTR	NM_000371.4	NP_000362.1	c.245A>T	p.E82V	3
chr18	TTR	NM_000371.4	NP_000362.1	c.254T>C	p.V85A	3
chr18	TTR	NM_000371.4	NP_000362.1	c.256G>C	p.E86Q	3
chr18	TTR	NM_000371.4	NP_000362.1	c.257A>T	p.E86V	3
chr18	TTR	NM_000371.4	NP_000362.1	c.258A>T	p.E86D	3
chr18	TTR	NM_000371.4	NP_000362.1	c.259G>C	p.G87R	3
chr18	TTR	NM_000371.4	NP_000362.1	c.25C>A	p.L9I	3
chr18	TTR	NM_000371.4	NP_000362.1	c.286A>G	p.K96E	3
chr18	TTR	NM_000371.4	NP_000362.1	c.287A>G	p.K96R	3
chr18	TTR	NM_000371.4	NP_000362.1	c.287A>T	p.K96I	3
chr18	TTR	NM_000371.4	NP_000362.1	c.290C>G	p.S97C	3
chr18	TTR	NM_000371.4	NP_000362.1	c.290C>T	p.S97F	2
chr18	TTR	NM_000371.4	NP_000362.1	c.293A>C	p.Y98S	3
chr18	TTR	NM_000371.4	NP_000362.1	c.2T>A	p.M1K	3
chr18	TTR	NM_000371.4	NP_000362.1	c.300G>T	p.K100N	3
chr18	TTR	NM_000371.4	NP_000362.1	c.302C>T	p.A101V	3
chr18	TTR	NM_000371.4	NP_000362.1	c.311T>A	P.I104N	2
chr18	TTR	NM_000371.4	NP_000362.1	c.316C>A	p.P106T	3
chr18	TTR	NM_000371.4	NP_000362.1	c.317C>G	p.P106R	3

chr18	TTR	NM_000371.4	NP_000362.1	c.319T>G	p.F107V	3
chr18	TTR	NM_000371.4	NP_000362.1	c.320T>C	p.F107S	3
chr18	TTR	NM_000371.4	NP_000362.1	c.321C>G	p.F107L	3
chr18	TTR	NM_000371.4	NP_000362.1	c.322C>G	p.H108D	3
chr18	TTR	NM_000371.4	NP_000362.1	c.324T>A	p.H108Q	3
chr18	TTR	NM_000371.4	NP_000362.1	c.325G>A	P.E109K	2
chr18	TTR	NM_000371.4	NP_000362.1	c.327G>T	p.E109D	3
chr18	TTR	NM_000371.4	NP_000362.1	c.334G>C	p.E112Q	3
chr18	TTR	NM_000371.4	NP_000362.1	c.335A>G	p.E112G	3
chr18	TTR	NM_000371.4	NP_000362.1	c.336G>T	p.E112D	3
chr18	TTR	NM_000371.4	NP_000362.1	c.349G>T	p.A117S	2
chr18	TTR	NM_000371.4	NP_000362.1	c.34G>A	p.A12T	3
chr18	TTR	NM_000371.4	NP_000362.1	c.34G>T	p.A12S	3
chr18	TTR	NM_000371.4	NP_000362.1	c.353A>G	p.N118S	3
chr18	TTR	NM_000371.4	NP_000362.1	c.355G>A	p.D119N	3
chr18	TTR	NM_000371.4	NP_000362.1	c.356A>C	p.D119A	3
chr18	TTR	NM_000371.4	NP_000362.1	c.370C>T	p.R124C	3
chr18	TTR	NM_000371.4	NP_000362.1	c.371G>A	p.R124H	3
chr18	TTR	NM_000371.4	NP_000362.1	c.38G>T	p.G13V	3
chr18	TTR	NM_000371.4	NP_000362.1	c.401A>G	p.Y134C	2
chr18	TTR	NM_000371.4	NP_000362.1	c.424G>A	p.V142I	2
chr18	TTR	NM_000371.4	NP_000362.1	c.430A>G	p.N144D	3
chr18	TTR	NM_000371.4	NP_000362.1	c.434C>G	p.P145R	3

chr18	TTR	NM_000371.4	NP_000362.1	c.43G>A	p.V15I	3
chr18	TTR	NM_000371.4	NP_000362.1	c.440A>C	p.E147A	3
chr18	TTR	NM_000371.4	NP_000362.1	c.441A>C	p.E147D	3
chr18	TTR	NM_000371.4	NP_000362.1	c.62G>C	p.G21A	3
chr18	TTR	NM_000371.4	NP_000362.1	c.62G>T	p.G21V	3
chr18	TTR	NM_000371.4	NP_000362.1	c.65C>A	p.P22H	3
chr18	TTR	NM_000371.4	NP_000362.1	c.67A>C	p.T23P	3
chr18	TTR	NM_000371.4	NP_000362.1	c.70G>T	p.G24C	3
chr18	TTR	NM_000371.4	NP_000362.1	c.71G>C	p.G24A	3
chr18	TTR	NM_000371.4	NP_000362.1	c.71G>T	p.G24V	3
chr18	TTR	NM_000371.4	NP_000362.1	c.73A>G	p.T25A	3
chr18	TTR	NM_000371.4	NP_000362.1	c.73A>T	p.T25S	3
chr18	TTR	NM_000371.4	NP_000362.1	c.74C>A	p.T25N	3
chr18	TTR	NM_000371.4	NP_000362.1	c.74C>T	p.T25I	3
chr18	TTR	NM_000371.4	NP_000362.1	c.76G>A	p.G26S	3
chr18	TTR	NM_000371.4	NP_000362.1	c.83C>A	p.S28Y	3
chr18	TTR	NM_000371.4	NP_000362.1	c.8C>G	p.S3C	3
chr18	TTR	NM_000371.4	NP_000362.1	c.98T>C	p.M33T	3
